# Protein-protein interaction analysis of Alzheimer`s disease and NAFLD based on systems biology methods unhide common ancestor pathways 

**Published:** 2018

**Authors:** Reza Karbalaei, Marzieh Allahyari, Mostafa Rezaei-Tavirani, Hamid Asadzadeh-Aghdaei, Mohammad Reza Zali

**Affiliations:** 1 *Proteomics Research Center, Faculty of Paramedical Sciences, Shahid Beheshti University of Medical Sciences, Tehran, Iran*; 2 *Drug Design and Bioinformatics Unit, Medical Biotechnology Department, Biotechnology Research Center, Pasteur Institute of Iran, Tehran, Iran*; 3 *Basic and Molecular Epidemiology of Gastrointestinal Disorders Research Center, Research Institute for Gastroenterology and Liver Diseases, Shahid Beheshti University of Medical Sciences, Tehran, Iran *; 4 *Gastroenterology and Liver Diseases Research Center, Research Institute for Gastroenterology and Liver Diseases, Shahid Beheshti University of Medical Sciences, Tehran, Iran*

**Keywords:** Alzheimer`s disease (AD), Non-alcoholic fatty liver disease (NAFLD), Protein-protein interaction (PPI) network analysis, Hub-bottlenecks, Protein clusters

## Abstract

**Aim:**

Analysis reconstruction networks from two diseases, NAFLD and Alzheimer`s diseases and their relationship based on systems biology methods.

**Background:**

NAFLD and Alzheimer`s diseases are two complex diseases, with progressive prevalence and high cost for countries. There are some reports on relation and same spreading pathways of these two diseases. In addition, they have some similar risk factors, exclusively lifestyle such as feeding, exercises and so on. Therefore, systems biology approach can help to discover their relationship.

**Methods:**

DisGeNET and STRING databases were sources of disease genes and constructing networks. Three plugins of Cytoscape software, including ClusterONE, ClueGO and CluePedia, were used to analyze and cluster networks and enrichment of pathways. An R package used to define best centrality method. Finally, based on degree and Betweenness, hubs and bottleneck nodes were defined.

**Results:**

Common genes between NAFLD and Alzheimer`s disease were 190 genes that used construct a network with STRING database. The resulting network contained 182 nodes and 2591 edges and comprises from four clusters. Enrichment of these clusters separately lead to carbohydrate metabolism, long chain fatty acid and regulation of JAK-STAT and IL-17 signaling pathways, respectively. Also seven genes selected as hub-bottleneck include: IL6, AKT1, TP53, TNF, JUN, VEGFA and PPARG. Enrichment of these proteins and their first neighbors in network by OMIM database lead to diabetes and obesity as ancestors of NAFLD and AD.

**Conclusion:**

Systems biology methods, specifically PPI networks, can be useful for analyzing complicated related diseases. Finding Hub and bottleneck proteins should be the goal of drug designing and introducing disease markers.

## Introduction

 Alzheimer’s disease (AD) is a neurodegenerative disease that is one of the important disease in industrial countries. Based on Alzheimer’s Disease International Federation (ADI),at least 46.8 million people are affected by dementia worldwide , that anticipated to be 74.7 million by 2030 and 131.5 million by 2050 (1). This disease can be categorized in two forms: early-onset familial Alzheimer disease (EFAD) (2) and Late-onset Alzheimer’s disease (LOAD) or non-familial (3). EFAD form inheritance dominantly but LOAD form is a complex or multifactorial disease (4). Research on AD showed that in addition to age and heredity, lifestyle is an important factor in the progression of this disease (5). On the other hand, lifestyle has an important role in producing some diseases such as obesity, diabetes and fatty liver (6). Fatty liver diseases divided into two forms : alcoholic fatty liver disease (AFLD) and Non-alcoholic fatty liver disease (NAFLD), that mainly occurs due to high using of Alcohol and fat (6). Non-alcoholic fatty liver disease (NAFLD) is one of the most important reasons for liver disease in the United States so that 30% of US population affected by NAFLD (7). Indeed, as well as AD, NAFLD depend on lifestyle and feeding. 

Our previous studies on AD that accomplished by meta-analysis in microarray data showed that NAFLD has an undoubted relation to AD (8). There are other studies about the relation of AD and NAFLD that focuses on some common genes (LRP1) (9), cross-sectional study (10) and AD-Transgenic model (11). Protein-protein interaction (PPI) network analysis is one of the major fields in systems biology in which analyzed complex interactome of proteins as the main source of data (12). Using systems biology method such as a comparison between gene sets of diseases, constructing PPI network and pathway enrichment can be helpful to decipher the shared mechanism of NAFLD and AD. In this study, we reported seven important shared proteins between these diseases that can be used not only as markers of disease but also as targets for drug designing. Also, pathways that shred between these diseases were introduces. 

## Methods

DisGeNET is a discovery database that gathered genes and variants associated with human diseases and publicly available (13). The related genes of NAFLD and AD were exported from DisGeNET database and common genes between two diseases used to construct PPI network by Search Tool for the Retrieval of Interacting Genes/Proteins (STRING). STRING is a database for predicted protein-protein interactions at EMBL clusters the extracted results from many protein-protein interactions databases, like Mint, BioGrid, etc. It also uses the information from KEGG pathways and Reactome to provide the best annotations for the interactions of one protein (14). The common network was constructed by importing shared genes in STRING database and clustered by ClusterONE plugin of Cytoscape software (15) that finds overlapping protein complexes in a protein interaction network loaded into Cytoscape. (overlap threshold = 1, node penalty = 0, haircut threshold = 0) (16). Pathway enrichment and the relation between pathways were accomplished using ClueGO and CluePedia plugins of Cytoscape software (17, 18). To find the best centrality method for selection of the most important nodes, we use an R package named CINNA (19, 20). A network is composed of nodes (e.g., genes or proteins) and edges/links (e.g., co-expression relationships or physical interactions). In network biology terms, degree, and Betweenness are important centrality parameters that are useful for analysis network topology. Edges/links of a node are called the degree of that node. Nodes with high degree are called hubs and nodes that achieve top-ten, or top-five percent of betweenness centrality are called bottlenecks (both based on researcher’s definition) (21). So, nodes that are simultaneously hubs and bottlenecks are named hub-bottlenecks (22). Average degree (A.D) and standard deviation (SD) of degrees were calculated, and nodes with a degree above 2SD + A.D were selected as hub proteins in each network. Also, the top five percent of betweenness centrality measures were selected as bottleneck proteins. Shared genes, hubs and bottleneck proteins of these two networks were extracted and used for further analysis. We used Cytoscape to analyze networks and extract hubs, hub-bottlenecks, and their first neighbors (23). 

**Figure 1:**
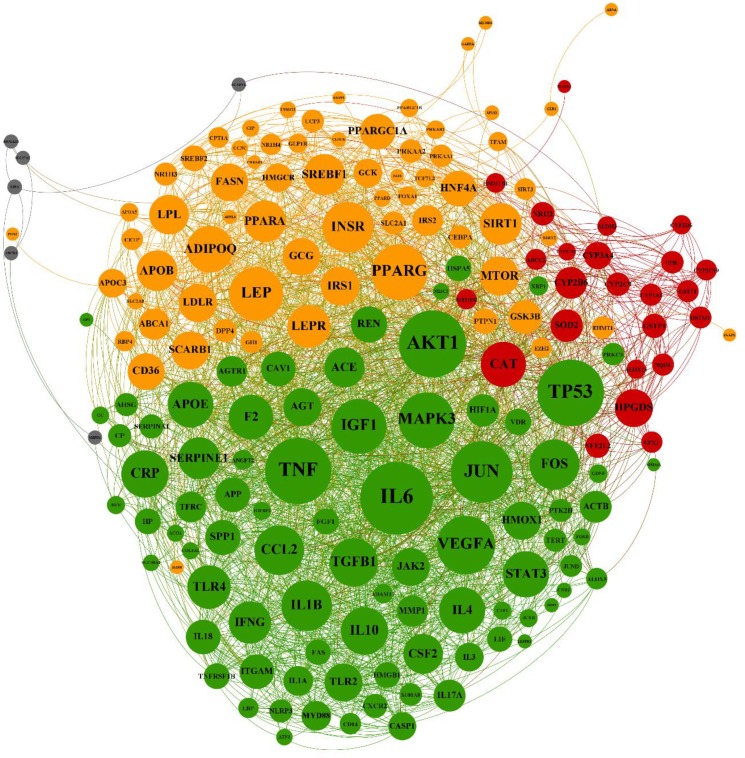
Resulted network which is constructed by common genes between NAFLD and AD diseases is presented. This network includes four clusters that are highlighted by different colors. Cluster-1: orange, cluster-2: red, cluster-3: green, and cluster-4: gray

**Table 1:** List of enriched pathways based on network clustering related to the common genes between NAFLD and AD is presented. Cluster four does not have has pathways, due to less number of genes

GO Term	Ontology Source	Adjusted-P Value	Cluster name
regulation of glucose import	GO Biological Process	1.1E-11	1
glucose import	GO Biological Process	4.5E-14	
AMPK signaling pathway	KEGG	1.3E-25	
Longevity regulating pathway	KEGG	5.1E-11	
Adipocytokine signaling pathway	KEGG	1.7E-20	
fatty acid oxidation	GO Biological Process	1.4E-14	
PPAR signaling pathway	KEGG	3.1E-13	
Adipocytokine signaling pathway	KEGG	1.7E-20	
Insulin resistance	KEGG	5.8E-23	
regulation of fatty acid metabolic process	GO Biological Process	4.2E-20	
positive regulation of fatty acid metabolic process	GO Biological Process	1.3E-14	
fatty acid beta-oxidation	GO Biological Process	1.4E-11	
long-chain fatty acid metabolic process	GO Biological Process	1.2E-16	2
arachidonic acid metabolic process	GO Biological Process	4.3E-16	
Metabolism of xenobiotics by cytochrome P450	KEGG	5.1E-23	
Drug metabolism	KEGG	3.3E-23	
Chemical carcinogenesis	KEGG	6.9E-20	
Oxidation by Cytochrome P450	WikiPathways	6.9E-16	
Vitamin D (calciferol) metabolism	REACTOME	7.5E-16	
Cytochrome P450 - arranged by substrate type	REACTOME	7.5E-16	
Vitamins	REACTOME	7.5E-16	
Oncostatin M Signaling Pathway	WikiPathways	1.4E-28	3
Lung fibrosis	WikiPathways	1.5E-24	
AGE-RAGE signaling pathway in diabetic complications	KEGG	6.2E-28	
Interleukin-10 signaling	REACTOME	6E-48	
Interleukin-4 and 13 signaling	REACTOME	6E-48	
positive regulation of JAK-STAT cascade	GO Biological Process	4.3E-23	
tyrosine phosphorylation of STAT protein	GO Biological Process	2E-21	
regulation of tyrosine phosphorylation of STAT protein	GO Biological Process	1.5E-21	
IL-17 signaling pathway	KEGG	2.1E-26	
Salmonella infection	KEGG	7.8E-27	
Pertussis	KEGG	1.4E-25	
Leishmaniasis	KEGG	6.5E-28	
Chagas disease (American trypanosomiasis)	KEGG	7.4E-30	
Inflammatory bowel disease (IBD)	KEGG	1.7E-26	
Rheumatoid arthritis	KEGG	1.3E-28	
Allograft Rejection	WikiPathways	1.2E-21	
lipopolysaccharide-mediated signaling pathway	GO Biological Process	4.6E-27	

**Table 2:** Hub and Bottleneck genes of Common network genes between NAFLD and AD are shown. Hub-bottleneck genes are bolded

R	Hubs	Degree	Bottlenecks	Betweenness score
1	IL6	109	TP53	0.087078
2	AKT1	99	IL6	0.06467
3	TP53	98	AKT1	0.054262
4	TNF	97	TNF	0.047973
5	JUN	89	JUN	0.034409
6	VEGFA	83	PPARG	0.030972
7	PPARG	79	INSR	0.026792
8	MAPK3	78	VEGFA	0.024631
9	IGF1	77	LPL	0.021859
10	LEP	75	CAT	0.020219

**Table 3:** OMIM result from enrichment of Hub-bottleneck genes and their first neighbors are represented

disease name	Adjusted P-value	Genes
diabetes	3.41E-10	TCF7L2;IL6;ACE;IRS1;HFE;HNF4A;INSR;UCP3;IRS2;PPARG;GCK
diabetes_mellitus,_type_2	1.35E-09	TCF7L2;IRS1;HNF4A;INSR;UCP3;IRS2;PPARG;GCK
obesity	3.87E-05	LEP;UCP3;LEPR;PPARG;PPARGC1B
diabetes_mellitus	4.25E-05	TCF7L2;IRS1;HNF4A;INSR;IRS2;GCK

## Results

From DisGeNET, 332 and 1200 genes were extracted for NAFLD and Alzheimer`s diseases, respectively. Totally, 189 genes were shared between the two lists were shared and were named common genes. The common genes network that was constructed using STRING database has 182 nodes and 2591 edges and four clusters (Figure 1). Cluster analyzing by ClueGO and CluePedia plugins showed that there are 29 meaningful pathways based on statistical analysis and there is no duplication between them. Cluster one mainly includes carbohydrate metabolism pathways and their related signaling, and the main category of this cluster was AMPK signaling pathway. In cluster two long chain fatty acid and their extract metabolic process include arachidonic acid, xenobiotics and calciferol were enriched. Finally, enrichment of cluster three lead to signaling pathways such as regulation of JAK-STAT cascade, IL-17 signaling pathway and AGE-RAGE signaling pathway in diabetic complications. Due to low number of nodes in cluster four, pathway enrichment was meaningless (table 1). 

Based on CINNA package results, degree and Betweenness centrality methods were the best qualified methods for this network. In next step, the network was analyzed by Cytoscape to define hubs, hub-bottleneck. Results showed that IL6, AKT1, TP53, TNF, JUN, VEGFA, PPARG, MAPK3, IGF1, and LEP are hubs that first seven proteins were also bottlenecks, so selected as hub-bottlenecks (table 2). By extracting these hub-bottlenecks and their first neighbors from the network, we reach to a new interesting network that contains 82% of nodes (150 nodes) and 92% of edges (2367 edges) from the main network. So by analyzing them in OMIM as the main database for disease, we reached to diabetes and obesity (table 3).

## Discussion

Systems biology methods such as PPI network analysis and pathway enrichment have been used broadly to discover main proteins and pathways underlying complex diseases (24). Different types of cancers, various kinds of neurodegenerative diseases and disorders and also many cellular conditions are analyzed via protein-protein interaction method (25-30) The relation between NAFLD and AD is becoming increasingly recognized (9-11). In this study, we used the complete genes list of the two diseases (NAFLD and AD) that may have shared mechanism based on risk factors and previous studies (8). According to network clustering and further pathways enrichment, 42 pathways were enriched. Altogether, three main group of pathways are candidate as key pathways in both AD and NAFLD: carbohydrate metabolism, long fatty acid metabolism, and IL signaling pathways. Previous studies indicate evidence about all mentioned relation except the role of long fatty acid metabolism in AD (8, 31-37). 

Six Hub- Bottleneck nodes are important targets for both NAFLD and AD. High level secretion of peripheral IL-6 may be responsible for acute-phase proteins that observed in AD patients (38) and high levels of IL-6 were detected in NAFLD patients (39). AKT activity in temporal cortex of Alzheimer patients were significantly increased (40) and activated the PI3-K/Akt kinase pathway triggers NAFLD (41). TP53 that known as P53, up-regulated in Alzheimer's disease (42) and inhibition of attenuates signs of NAFLD (43). Inhibition of TNF alpha decrease amyloid plaques and tau phosphorylation in the mouse brain, and so risk of AD (44), and this protein involved in the pathophysiology of NAFLD (45). Inhibition of JUN is a therapeutic strategy to stop progression of AD (46) and expression of this protein Increased in NAFLD (47). Abnormal regulation of VEGFA expression implicated in AD (48) and involved in pathophysiology of NAFLD (49). Finally, PPARG is a potential therapeutic targets for both AD and NAFLD (50, 51). 

Analyzing main nodes and their first neighbors by OMIM database showed that diabetes and obesity were the results of this enrichment. We can conclude that diabetes and obesity are common ancestors of AD and NAFLD. 

These results showed that application of systems biology methods unhide unravels the secret behind common mechanism of AD and NAFLD. The real impact of common proteins on treatment of NAFLD and AD also needs to be further assessed.

## Conflict of interests

The authors declare that they have no conflict of interest.
